# Plasticity of intrinsic excitability during LTD is mediated by bidirectional changes in h-channel activity

**DOI:** 10.1038/s41598-017-14874-z

**Published:** 2017-10-31

**Authors:** Célia Gasselin, Yanis Inglebert, Norbert Ankri, Dominique Debanne

**Affiliations:** UNIS, INSERM U-1072, Aix-Marseille University, Marseille, France

## Abstract

The polarity of excitability changes associated with induction of Long-Term synaptic Depression (LTD) in CA1 pyramidal neurons is a contentious issue. Postsynaptic neuronal excitability after LTD induction is found to be reduced in certain cases (i.e. synergistic changes) but enhanced in others (i.e. compensatory or homeostatic). We examined here whether these divergent findings could result from the activation of two separate mechanisms converging onto a single learning rule linking synergistic and homeostatic plasticity. We show that the magnitude of LTD induced with low frequency stimulation (LFS) of the Schaffer collaterals determines the polarity of intrinsic changes in CA1 pyramidal neurons. Apparent input resistance (R_in_) is reduced following induction of moderate LTD (<20–30%). In contrast, R_in_ is increased after induction of large LTD (>40%) induced by repetitive episodes of LFS. The up-regulation of *I*_h_ observed after moderate LTD results from the activation of NMDA receptors whereas the down-regulation of *I*_h_ is due to activation of mGluR1 receptors. These changes in R_in_ were associated with changes in intrinsic excitability. In conclusion, our study indicates that changes in excitability after LTD induction follow a learning rule describing a continuum linking synergistic and compensatory changes in excitability.

## Introduction

In central neurons, changes in intrinsic neuronal excitability have been shown to occur in parallel with synaptic modifications, thus affecting synergistically synaptic strength and dendritic integration in the post-synaptic neuron^1,2^. Induction of Long-Term synaptic Potentiation (LTP) is associated with an increase in the firing probability of the postsynaptic neuron^3–7^, whereas induction of Long-Term synaptic Depression (LTD) is associated with a reduced firing probability in response to the test input^4,5,8^.

Induction of synaptic plasticity has also been associated with non-synergistic (i.e. homeostatic) modifications of intrinsic excitability^9,10^. One of the key players in CA1 pyramidal cells is the hyperpolarization-activated cationic h-current (*I*_h_), a major determinant of input resistance and intrinsic neuronal excitability^11,12^. Very large LTP (≈+300%) was found to up-regulate *I*_h_ to counteract excessive synaptic excitation^13,14^ whereas large LTD (≈−60%) was found to down-regulate *I*_h_ to counteract excessive synaptic depression^15^.

These two homeostatic regulations of *I*_h_ are not compatible with the synergistic changes in excitability and synaptic strength reported earlier^4,5,16^. The discrepancy for the LTP side was resolved by showing that h-channel regulation depended on LTP amplitude^17^. In fact, it was shown in this study that physiological LTP (i.e. +20–50%) produced a *decrease* in *I*_h_ seen as an increase in R_in_ whereas extreme LTP (i.e. +200–300%) produced an *increase* in *I*_h_ and the two extrema were linked by a continuum of synergistic and homeostatic plasticity. However, the discrepancy still remained for LTD. We examined whether a similar continuum also exists for homeostatic and synergistic changes in intrinsic neuronal excitability for the LTD side.

We show here that R_in_ also depends on the magnitude of LTD, with a decrease following induction of moderate LTD but an increase after induction of strong LTD. This dependence of R_in_ on the magnitude of LTD is abolished by the h-channel blocker ZD-7288. The decrease in R_in_ (due to an up-regulation of *I*_h_) is mediated by NMDA receptor activation whereas the increase in R_in_ (i.e. due to a down-regulation of *I*_h_) is mediated by activation of mGluR1. We show here that induction of LTD in the presence of the NMDA receptor antagonist D-AP5 enhanced neuronal excitability whereas LTD induction in the presence of the mGluR antagonist LY341485 diminished excitability of CA1 pyramidal neurons. We conclude that intrinsic plasticity induced by LTD also describes a continuum between synergistic and homeostatic plasticity in CA1 pyramidal neurons, involving different sets of glutamate receptors.

## Results

### LTD magnitude determines changes in R_in_ in CA1 pyramidal neurons

**Figure 1:**
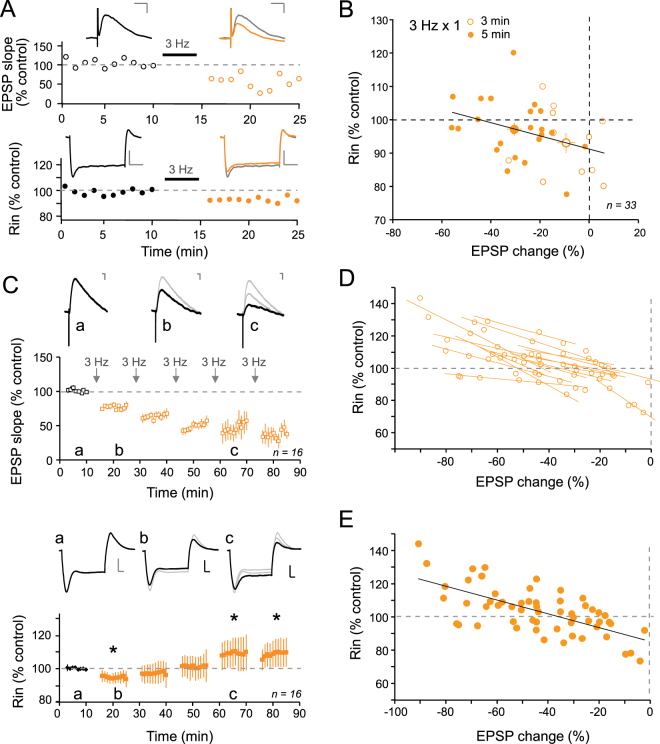
LTD magnitude determines changes in R_in_. (**A**) Time-courses of EPSP slope (top) and R_in_ (bottom) in a single experiment showing a reduction in R_in_ following induction of LTD. Scale bars: top, 2 mV & 20 ms; bottom, 5 mV & 200 ms. (**B**) Plot of R_in_ as a function of EPSP change induced by 3 Hz stimulation for 3 or 5 minutes. Note the negative correlation (y = 0.198x + 91.096, r = 0.36; p < 0.01). (**C**) Top, EPSP slope time course pooled over sixteen experiments. Arrows indicate 3 Hz stimulation episodes. Representative EPSP traces in control (a), after the first stimulation episode (b) and after the third (c). Scale bars: 1 mV, 10 ms. Bottom, time course of apparent input resistance (R_in_) after each stimulation episode. R_in_ is reduced after the first 3 Hz stimulation (a, b) and increased for the third (c). Stars indicate statistical significance (p < 0.05) Top, representative R_in_ traces in control (a), after the first stimulation episode (b) and after the third (c). Scale bars: 10 mV, 100 ms. (**D**) Normalized R_in_ as a function of EPSP change for each cell. Note that correlations for each cell are all negatively oriented. (**E**) Normalized R_in_ changes versus normalized LTD level for each stimulation episode. A significant linear negative correlation was observed (y = −0.414x + 85.487; r = 0.66; p < 0.001).

All experiments were performed in the presence of the GABA receptor antagonist PiTx (100 µM). EPSPs were evoked in CA1 pyramidal neurons recorded in whole-cell configuration by stimulating the Schaffer collaterals at 0.1 Hz. After obtaining a stable base line, Long-Term Depression (LTD) of synaptic transmission was induced by stimulation of the Schaffer collaterals at 3 Hz during 3 or 5 min. Input resistance (R_in_) measured with large hyperpolarizing current pulses to recruit h-current (−120 pA, 800 ms) was found to be reduced to ~95.7% of the control value (n = 33, t-test p < 0.01) following LTD induction (Fig. 1A). But more interestingly a negative correlation was observed between the normalized R_in_ and the synaptic change (y = −0.198x-91.1; r = 0.36; p < 0.05; Fig. 1B). This negative correlation was further confirmed by the difference in the mean R_in_ change observed after 3 or 5 min at 3 Hz (after 3 min at 3 Hz: 93 ± 3%, n = 11 for a mean EPSP change of −9 ± 4%; after 5 min at 3 Hz: 97 ± 2% for an EPSP change of −31 ± 3%; Fig. 1B).

To confirm the correlation observed with one train of 3 Hz stimulation, synaptic depressions of larger magnitudes were induced by repeated episodes of 3 Hz stimulation with ten minutes intervals. A progressive decrease in EPSP slope and a parallel changes in apparent R_in_ were observed (Fig. 1C). While R_in_ decreased after the first stimulation episode (see also Fig. 1A), it progressively increased after each stimulation episode (Fig. 1C). The analysis of the trajectories of individual cells showed in all cases an anti-correlation (Fig. 1D). The plot of R_in_ versus EPSP change revealed a significant anti-correlation (r = 0.66; p < 0.001; Fig. 1E). R_in_ was reduced to 90 ± 3% for moderate LTD (<20%) but increased to 116 ± 4% for large LTD (>50%; Fig. 1E). As previously reported for LTP^17^, modulation of R_in_ was not associated with significant change in V_m_ following induction of LTD (−62.3 ± 0.9 mV in control and −61.8 ± 0.6 mV after the 3^rd^ episode of 3 Hz stimulation, p > 0.1; Supplementary Figure 1). In conclusion, the magnitude of LTD determines the polarity of R_in_ change in CA1 pyramidal neurons.

### Temporal stability of synaptic transmission and R_in_

**Figure 2:**
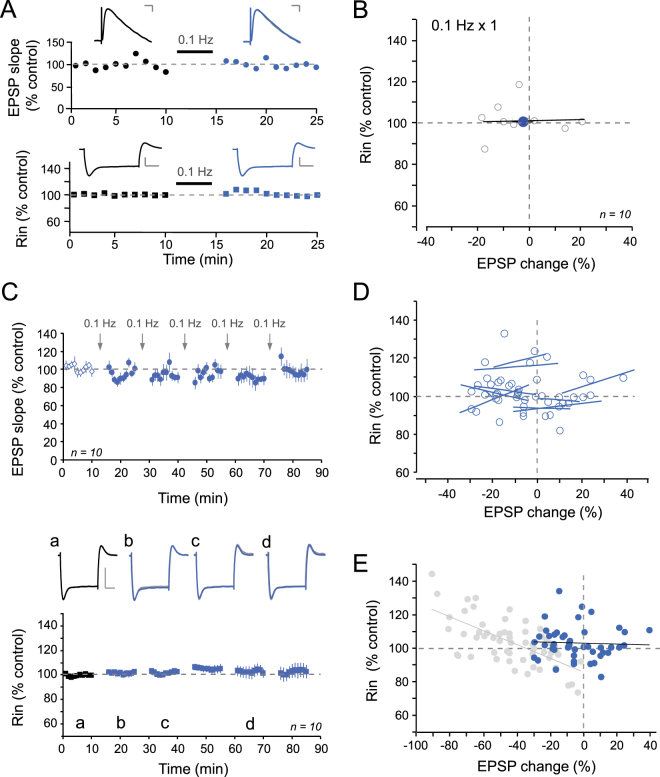
Temporal stability. (**A**) Time-courses of EPSP slope (top) and R_in_ (bottom) after 0.1 Hz stimulation. Scale bars; top, 2 mV & 20 ms; bottom, 10 mV & 200 ms. (**B**) Plot of R_in_ versus EPSP change. No correlation was observed (y = 0.021x + 101.31, r = 0.10). (**C**) Top, EPSP slope time-course pooled over ten experiments. Arrows indicate 0.1 Hz stimulation episodes. Bottom, time-course of R_in_. Representative R_in_ traces in control (a), and after stimulation episodes (b, c & d). Scale bars: 10 mV, 100 ms. No statistically significant changes were observed. (**D**) Correlation between R_in_ and EPSP change for each cell after 0.1 Hz stimulation. (**E**) Normalized R_in_ versus normalized EPSP change for all episodes of 0.1 Hz stimulation (blue dots). Compared to the control condition (grey dots, see Fig. 1B), no linear correlation was observed (y = −0.027x + 102.79; r = 0.05, p > 0.05).

In order to eliminate any non-specific R_in_ changes, we repeated the same protocol with 0.1 Hz stimulation to test the temporal stability of synaptic strength and R_in_ in CA1 pyramidal neurons. No changes in EPSP slope were observed after 1 (Fig. 2A & B) or several repetitive episodes of 0.1 Hz stimulation (−4 ± 3% of control EPSP slope; Fig. 2C). Furthermore R_in_ remained unchanged throughout the experiment (103 ± 1%; Fig. 2C). Finally, no linear correlation was observed between normalized R_in_ and EPSP slope at the level of individual cells (Fig. 2D) or all taken together (r = 0.05; p > 0.05; Fig. 2E).

### Regulation of *I*_h_ is responsible for changes in R_in_

**Figure 3:**
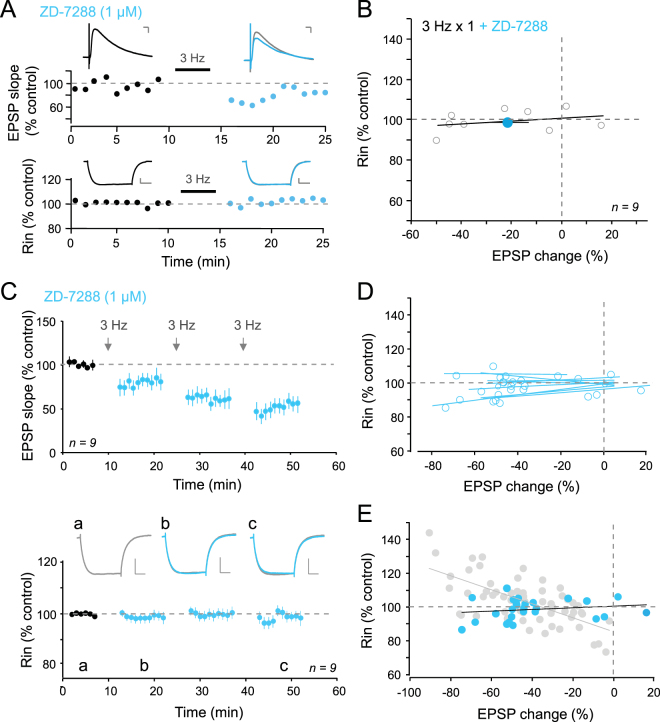
Blockade of h-channels ZD-7288 impairs regulation of R_in_. (**A**) Time-courses of EPSP slope (top) and R_in_ (bottom) following 3 Hz stimulation for 5 min in the presence of ZD-72288. Scale bars: top, 2 mV & 20 ms; bottom, 5 mV & 200 ms. (**B**) Plot of R_in_ as a function of EPSP change. Note the lack of correlation (y = 0.072 + 100.61; r = 0.31). (**C**) Top, EPSP slope time-course pooled over nine experiments with ZD7288 (1 µM) in the bath. Note that ZD7288 does not alter synaptic plasticity. Bottom, R_in_ time course. Representative traces in control (a), after the first stimulation episode (b) and after the third (c). Scale bars: 10 mV, 100 ms. No R_in_ changes were observed. (**D**) Negative correlations between R_in_ changes and LTD levels for each cell in the presence of ZD-7288. (**E**) Normalized R_in_ versus EPSP change levels in control (grey dots) and in the presence of ZD-7288 (blue dots). No significant linear correlation was observed (y = 0.05x + 100.6; r = 0.18; p > 0.05).

R_in_ is mainly governed by the h-current in CA1 pyramidal neurons. We therefore tested the role of *I*_h_ in the observed changes in R_in_. We repeated the same protocol in the presence of the pharmacological blocker of h-channels ZD-7288 (1 µM). This concentration of ZD-7288 has been shown to block *I*_h_ without altering excitatory synaptic transmission^18^. In the presence of ZD-7288, stimulation of the Schaffer collaterals at 3 Hz still induced LTD (−23 ± 9%, n = 8; Fig. 3A) but R_in_ remained unchanged (98 ± 2% of control R_in_, n = 8, Fig. 3A & B). Similarly, no change in R_in_ occurred following induction of incremental LTD by repeated low frequency stimulation at 3 Hz (Fig. 3C & D). No linear correlation was found between LTD magnitude and R_in_ changes in the presence of ZD-7288 (r = 0.18; p > 0.05; Fig. 3E). These results indicate that *I*_h_ is directly involved in the bidirectional regulation of R_in_ following induction of LTD.

To further confirm the implication of h-channels, the sag produced by activation of *I*_h_ was analysed. The sag was found to decrease after the 1^st^ episode of 3 Hz stimulation and remained reduced by ~15% thereafter (Supplementary Figure 2A). Interestingly, a significant correlation between the normalized sag change and the magnitude of LTD was observed (Supplementary Figure 2B). But, surprisingly, no increase in the sag amplitude was observed following induction of LTD. We thus developed a simplified model of hippocampal neuron in which h conductance (G_h_) increased from 0 to 10 nS (Supplementary Figure 2C). Importantly, while R_in_ provided a good description of changes in G_h_, the sag increased when G_h_ increased in the 0–2 nS range but it was found to decrease when G_h_ increased in the 2–10 nS range. This result indicates that the sag is a not an index appropriate for evaluating activity-dependent regulation of h-channels.

### Reduction of R_in_ depends on NMDAR

**Figure 4:**
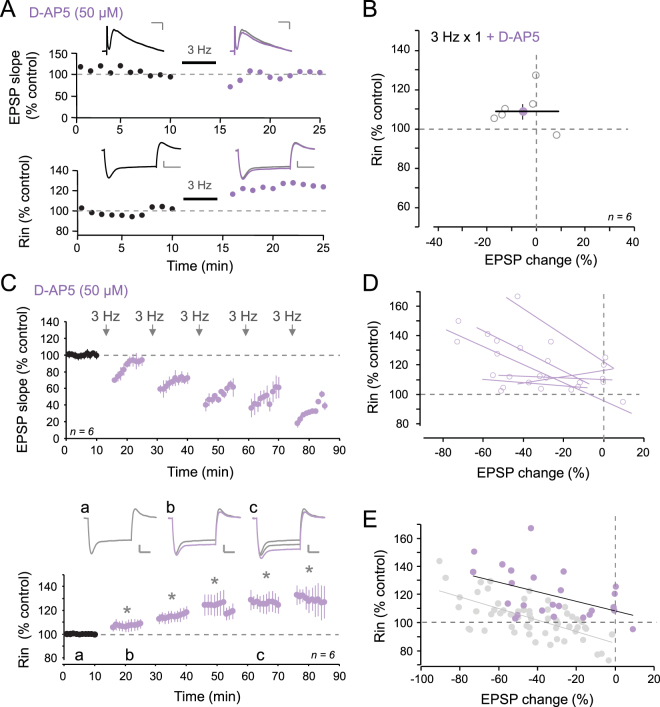
Blockade of NMDAR prevents decrease in R_in_. (**A**) Time-courses of EPSP slope (top) and R_in_ (bottom) following 3 Hz stimulation for 5 min in the presence of D-AP5. Scale bars: top, 2 mV & 20 ms; bottom, 5 mV & 200 ms. (**B**) Plot of R_in_ as a function of EPSP change. (**C**) Top, EPSP slope time-course induced by repetitive 3 Hz stimulation in the presence of 50 µM D-AP5 in the bath. Bottom, corresponding R_in_ time-course after each stimulation episode. R_in_ is increased from the first episode to the last one. Stars indicate statistical significance (p < 0.05). Representative R_in_ traces in control (a), after the first stimulation episode (b) and after the third (c). Scale bars: 10 mV, 100 ms. (**D**) Individual linear correlations between R_in_ changes and EPSP slope modifications induced by 3 Hz stimulation in the presence of D-AP5. (**E**) Normalized R_in_ changes versus normalized LTD size for control condition (grey dots) and in presence of D-AP5 (purple dots). Note the shift to higher values of R_in_ when D-AP5 is present during the plasticity induction (correlation: y = −0.34x + 108.7, r = 0.47; p < 0.05).

Induction of LTD requires both N-methyl-D-aspartate receptor (NMDAR)^19–21^ and/or metabotropic glutamate receptor (mGluR)^15,22–24^. To dissect the role of NMDAR in the regulation in R_in_, we applied the specific antagonist D-AP5 (50 µM) in the bath. In the presence of D-AP5, the magnitude of LTD induced by the first episode of 3 Hz stimulation was found to be reduced (93 ± 7% of control EPSP slope, n = 6 versus 72 ± 4%, n = 16, in control condition; Fig. 4A & B). In contrast with what was observed in control conditions, R_in_ was increased in 5 out of 6 cells after the first stimulation episode (110 ± 4% of control R_in_, Fig. 4A & B).

The following stimulation episodes produced, however, comparable levels of LTD and R_in_ was found to augment up to 130% after the last episode of 3 Hz stimulation (128 ± 1%; Fig. 4C & D). Compared to the control situation, the plot of normalized R_in_ against EPSP change in D-AP5 indicates an upward shift of the linear anti-correlation (r = 0.47; p < 0.05; Fig. 4E). These data suggest that NMDARs are implicated in the down-regulation of R_in_ observed for moderate LTD. The remaining increase in R_in_ might result from the stimulation of mGluRs.

### Enhancement of R_in_ depends on mGluR1

**Figure 5:**
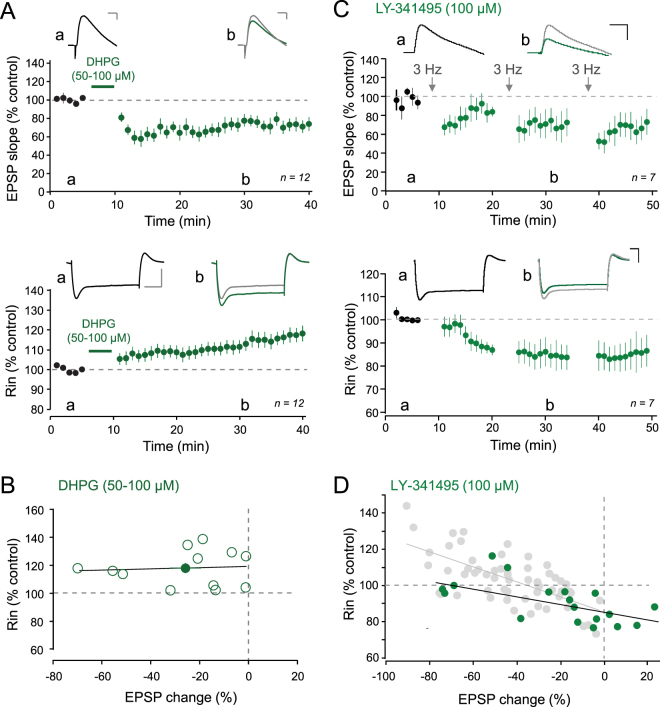
Blockade of mGluRs prevents increase in R_in_. (**A**) Top, time-course of synaptic changes induced by bath application of 50–100 µM DHPG during 5 minutes (pooled data from 12 cells). Upper traces, representative examples of EPSPs before and after DHPG. Scale bars: top, 2 mV & 20 ms. Bottom, normalized R_in_ changes induced by DHPG. Upper traces, representative traces. Scale bars: 10 mV, 100 ms. (**B**) Plot of R_in_ as a function of EPSP changes. No correlation is observed (r = 0.06). (**C**) Top, time-course of synaptic changes induced by 3 Hz stimulation in the presence of 100 µM LY341495. Upper traces, representative examples of EPSPs before and after 3 Hz stimulation. Scale bars: 5 mV & 30 ms. Bottom, normalized R_in_ changes induced by 3 Hz stimulation in the presence of 100 µM LY341495. Scale bars: 10 mV & 50 ms. (**D**) Plot, of R_in_ as a function of EPSP changes (linear correlation, y = −0.203x + 85.1, r = 0.64; p < 0.01).

We next tested whether mGluRs were implicated in the up-regulation of R_in_. We first applied the mGluR1/5 agonist DHPG (50–100 µM) during 5 min^23^. DHPG induced synaptic LTD (74 ± 6% of the control EPSP slope, n = 12; Fig. 5A). Interestingly, this mGluR-induced LTD was associated with a long-lasting increase of R_in_ (116 ± 4% of the control R_in_; Fig. 5A & B).

Next, we induced LTD with 3 Hz stimulation of the Schaffer collaterals in the presence of the broad spectrum mGluR antagonist, LY341495 (100 µM). In this condition, synaptic LTD was still induced by 3 Hz stimulation (68 ± 12% of control EPSP slope, n = 7 after the 3^rd^ episode of stimulation; Fig. 5C) but importantly R_in_ was found to be reduced (to 85 ± 7% of control R_in_, n = 7 after the 3^rd^ episode of 3 Hz stimulation; Fig. 5C). Furthermore, the plot of normalized R_in_ against EPSP change in LY341495 was found to follow a linear anti-correlation (r = 0.64; Fig. 5D). In contrast, R_in_ was found to be still enhanced when LTD was induced in the presence of the specific mGluR5 antagonist, MPEP (10 µM; Supplementary Figure 3), suggesting that mGluR1 and not mGluR5 is responsible for the increase in R_in_.

In conclusion, the stimulation of NMDARs induces a decrease in R_in_ (i.e. up-regulation of *I*_h_) whereas the stimulation of mGluR1 is responsible for an increase in R_in_ (i.e. down-regulation of *I*_h_).

### Changes in excitability associated with LTD

**Figure 6:**
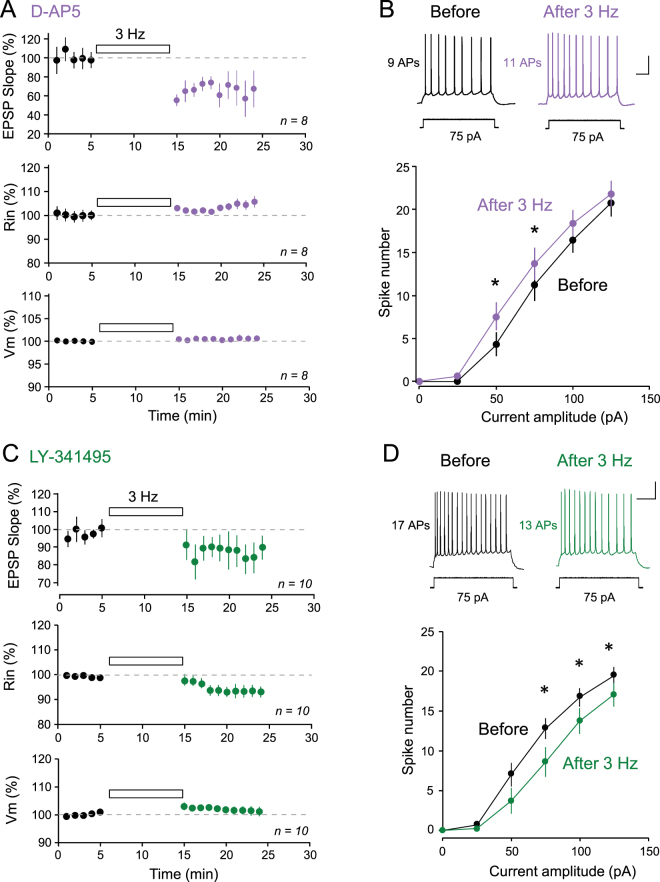
LTD is associated with bidirectional changes in excitability. (**A**) & (**B**) LTD induced in the presence of D-AP5 is associated with an increased excitability. (**A**) Top, time-course of LTD induced by 3 Hz stimulation of the glutamatergic inputs for 10 minutes in the presence of 50 µM D-AP5. Middle, time-course of the increased R_in_. Bottom, time-course of V_m_. (**B**) Top, representative example of firing induced by a current step of 75 pA before and after LTD induction with 3 Hz stimulation in the presence of D-AP5. Scale bars: 20 mV & 100 ms. Bottom, input-output curves before (black) and after (purple) LTD induction. Stars indicate significant change (p < 0.05). (**C**) & (**D**) LTD induced in the presence of LY341495 is associated with a decreased excitability. (**C**) Top, time-course of LTD induced by 3 Hz stimulation of the glutamatergic inputs for 10 minutes in the presence of 100 µM LY341495. Middle, time-course of the increased R_in_. Bottom, time-course of V_m_. (**D**) Top, representative example of firing induced by a current step of 75 pA before and after LTD induction with 3 Hz stimulation in the presence of LY341495. Scale bars: 20 mV & 200 ms. Bottom, input-output curves before (black) and after (dark green) LTD induction. Stars indicate significant change (p < 0.05).

Next, we tested whether these changes in Rin were associated with changes in intrinsic excitability following induction of LTD. To better dissect the implication of bidirectional changes in R_in_ we pharmacologically isolated the mGluR- and NMDAR-mediated component of R_in_ changes associated with LTD induced by 3 Hz stimulation of the Schaffer collateral for 10 min with either D-AP5 or LY341495 in the bath. Consistent with the increase in R_in_ after 3 Hz stimulation in the presence of D-AP5, excitability was found to be increased following LTD induction in D-AP5 (Fig. 6A & B). Conversely, in the presence of LY341495 excitability was found to be significantly reduced following induction of LTD (Fig. 6C & D).

In conclusion, LTD induced with 3 Hz stimulation activates NMDAR and mGluR that in turn regulate both R_in_ and intrinsic excitability in CA1 pyramidal cells.

## Discussion

**Figure 7:**
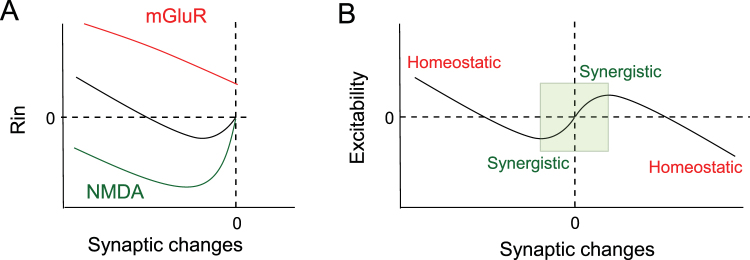
The continuum rule: linking synergistic plasticity with homeostatic plasticity. (**A**) Summary of the glutamate-receptor induced changes in R_in_ as a function of synaptic changes. Green curve illustrates the NMDA-dependent change in R_in_. Red curve shows the mGluR-dependent change in R_in_. Black curve illustrates the sum of the green and red curves. (**B**) Unifying rule for intrinsic plasticity. In a physiological range (defined by the green square), modulation of neuronal activity results in a conjugated modification in intrinsic excitability (synergistic). Out of this range, persisting increases (right) or decreases (left) in synaptic efficacy induce compensatory changes in intrinsic excitability (homeostatic). Adapted from Campanac *et al*. 2008 and from the present study.

We show here that in CA1 pyramidal neurons, LTD magnitude determines the changes in input resistance (R_in_) and hence, the direction of *I*_h_ regulation. Moderate LTD induces an increase in *I*_h_ (seen as a decrease in R_in_) while strong LTD results in a decrease of *I*_h_ (i.e. an increase in R_in_). LTD induction in the presence of the NMDA receptor antagonist D-AP5 suppressed the reduction in R_in_, suggesting that it is mediated by NMDA receptors (Fig. 7A). In contrast, LTD induced by activation of mGluR1/5 with DHPG is associated with an increase in R_in_ (i.e. decrease in *I*_h_). Furthermore, LTD induced in the presence of the mGluR antagonist LY341495 suppressed the increase in R_in_ and left it reduced by ~15%. However, no reduction in R_in_ was observed when LTD was induced in the presence of the mGluR5 antagonist, MPEP, suggesting that activation of mGluR1 and not mGluR5 triggers an increase in R_in_ (Fig. 7A). Finally, excitability was found to be increased when LTD was induced in the presence of D-AP5 whereas it was reduced when LTD was induced in the presence of LY341495. These results suggest that changes in intrinsic excitability follow a single learning rule linking synergistic changes induced by synaptic modification in the physiological range to homeostatic changes induced by large synaptic modification (Fig. 7B). Thus, our results bring strong evidence for fast compensatory processes in Hebbian plasticity^25^.

### LTD induces NMDAR-dependent up-regulation of *I*_h_

Our results show that a single episode of 3 Hz stimulation for 3–5 min decreases R_in_ in CA1 pyramidal neurons. Blocking *I*_h_ with ZD-7288 prevents changes in R_in_ following 3 Hz stimulation, indicating that R_in_ is decreased through an increase of *I*_h_. This component could be isolated by blocking mGluRs with LY341495. Because a reduction of R_in_ causes a decrease in intrinsic excitability^12^, this regulation is functionally synergic to the long-lasting depression of synaptic transmission. Such a Hebbian regulation of neuronal excitability has already been reported following LTD induction in CA1 neurons^4,5,16^, but this had not been reported in previous studies in which very large LTD was induced^15^. We show that in the presence of the NMDA receptor antagonist D-AP5, no decrease in R_in_ was observed. Rather, R_in_ was enhanced, indicating that stimulation of non-NMDA receptors triggers the down-regulation of h-channel activity.

### From Hebbian to homeostatic

Increasing LTD magnitude through repetition of 3 Hz stimulation episodes revealed that R_in_ could be regulated in the other direction. In fact, after the 3^rd^ or 4^th^ stimulation episode, large LTD was induced and R_in_ was found to be increased. This increase in R_in_ was prevented by the presence of ZD-7288 in the bath indicating that it was due to the down-regulation of *I*_h_. A reduction of *I*_h_ has already been demonstrated in CA1 pyramidal cells following LTD induction of large magnitude^15^. This regulation is supposed to counteract the reduction in synaptic efficiency in a homeostatic manner. In fact, other experimental studies have shown that sensory deprivation or chronic inactivity leads to the down-regulation of *I*_h_ in pyramidal neurons of the barrel cortex^26^ or the hippocampus^27^. The down-regulation of *I*_h_ could be induced by stimulation of group I mGluR. We indeed show that DHPG induced LTD associated with an increase in R_in_. In addition, we show that R_in_ diminished when LTD was induced by 3 Hz stimulation in the presence of the broad spectrum mGluR antagonist LY341495 but not in the presence of the specific mGluR5 antagonist MPEP, suggesting that activation of mGluR1 mediates the homeostatic increase in R_in_. These results are consistent with the mGluR-dependent increase in both R_in_ and intrinsic excitability reported by Brager & Johnson (2007) and suggest that two sets of receptors might be able to up-regulate and down-regulate h-channel activity depending on the magnitude of synaptic modification.

mGluR5 has been shown to mediate enhanced excitability induced by stimulation of glutamatergic inputs in L5 pyramidal neurons^28^ and in hippocampal parvalbumin-positive basket cells^7^. In these cases, the changes in excitability were synergistic to synaptic modification. Here, we show that stimulation of mGluR1 appears as the main factor responsible for the switch of synergistic to homeostatic regulation of intrinsic excitability.

Pharmacological^11^ or activity-dependent^15^ reduction in h-channel activity is usually associated with a hyperpolarizing shift in membrane potential. No change in membrane potential was, however, observed in the experiments reported here (see also Campanac *et al*., 2008). The apparent discrepancy with the results of Brager & Johnston (2007) might be due to the much larger increase in input resistance obtained in this study following LTD induction (+100% versus + 20% in our case).

Compared to Hebbian plasticity, homeostatic regulation is generally considered as a slow process. In fact, most of the regulations of intrinsic excitability reported so far have been obtained with manipulating network activity for 2–3 days^10,27,29–31^. Here, we report induction of homeostatic plasticity of intrinsic excitability that can be induced in parallel with Hebbian synaptic plasticity on a much faster time-scale. Such rapid compensatory processes are thought to be necessary to stabilize neuronal activity^32,33^.

Bidirectional regulation of *I*_h_ has already been revealed following LTP induction in CA1 pyramidal neurons^17^. The present study not only reconciles contradictive experimental results^4,15^ but it also shows that Hebbian and homeostatic regulations of *I*_h_ occur in the same neuron after LTD induction and follow a single rule establishing a continuum between functionally opposite forms of intrinsic plasticity that target h-channels (Fig. 7B)^34^.

### Mechanisms of h-channels regulation

The existence of a learning rule linking synergistic and homeostatic changes implies multiple modes of h-channel regulation. Although further experimental investigations will be required, the mechanisms of molecular regulation of h-channels are multiple^35^. Activity of h-channels can be regulated by a change in their density (i.e. by insertion or removal of HCN subunits), by a change in the distribution of h-channels at the surface of the neuron^36^ or by changes in their sensitivity to cyclic nucleotides^37^. Trip8b (Tetratricopeptide-Repeat containing Rab8b-interacting protein) has been identified as an important binding partner of HCN^38^. Interestingly, Trip8b undergo alternative splicing and its isoforms have been demonstrated to differently affect *I*_h_ density^39,40^ and the sensitivity of h-channels to cyclic AMP^40,41^. In fact, while most isoforms of Trip8b enhance expression of dendritic HCN subunits^39,40^, some Trip8b isoforms, however, suppress HCN subunit expression^42^. As dendrites are able to locally translate mRNA following LTD^43^ and promote alternative splicing^44^, Trip8b isoforms offer an attractive mechanism to explain the bidirectional regulation of *I*_h_. Interestingly, it has recently been shown that h-channel upregulation that normally occurs after induction of large LTP^13^ is absent in Trip8b knock-out mice^45^. Similar experiments should be conducted on the LTD side.

A remaining question is: what is the molecular link between activation of NMDAR/mGluR1 and the regulation of h-channels? The activation of different protein kinases such as Ca^2+^/CaMKII or PKC results in the modulation of h-channel activity in response to different patterns of neuronal activity^13,15,46^ but precise data on the regulation of Trip8b by either NMDAR or mGluR1 through Ca^2+^/CaMKII or PKC are still missing today.

## Experimental procedures

### Slice preparation

Hippocampal slices were obtained from 14- to 20- day-old rats according to institutional guidelines for the care and use of laboratory animals (Directive 86/609/EEC and French National Research Council) an approved by the local health authority (# D1305508, Préfecture des Bouches-du-Rhône, Marseille). Rats were deeply anaesthetized with chloral hydrate (intraperitoneal 400 mg/kg) and killed by decapitation. Slices (350 µm) were cut in a solution containing a reduced concentration of sodium (in mM: 280 sucrose, 26 NaHCO_3_, 10 D-glucose, 1.3 KCl, 1 CaCl_2_, and 10 MgCl_2_) on a vibratome (Leica VT1000S) and were maintained for 1 h at room temperature in oxygenated (95% O_2_/5% CO_2_) Artificial Cerebro-Spinal Fluid (ACSF; in mM: 125 NaCl, 2.5 KCl, 0.8 NaH_2_PO_4_, 26 NaHCO_3_, 3 CaCl_2_, 2 MgCl_2_, and 10 D-glucose) with foetal bovine serum (4%). For recording, each slice was transferred to a temperature-controlled (30 °C) chamber with oxygenated ACSF. GABA_A_ channels were blocked with picrotoxin (PiTX, 100 µM) and the CA3 area was surgically removed.

### Electrophysiology

Neurons were identified with an Olympus BX 50WI microscope using infrared video microscopy and Differential Interference Contrast (DIC) × 60 optics.

Whole-cell recordings were made from CA1 pyramidal neurons with electrodes filled with a solution containing the following (in mM): 120 K-gluconate, 20 KCl, 10 HEPES, 0.5 EGTA, 2 MgCl_2_ 6H_2_O, and 2 Na_2_ATP. Stimulating pipettes filled with extracellular saline were placed in the *stratum radiatum* to stimulate the Schaffer collaterals.

In control and test conditions, Excitatory Post-Synaptic Potentials (EPSPs) were elicited at 0.1 Hz by a digital stimulator (NEURO DATA PG4000, Instruments corp.) or by pCLAMP (Molecular devices). LTD was induced with continuous shocks delivered at 3 Hz during 5 min. Apparent input resistance was tested by current injection (−120 pA; 800 ms). Series resistance was monitored throughout the recording and only experiments with stable resistance were kept (changes <10%).

Intrinsic excitability has been measured before and after 3 Hz stimulation with input-output curves consisting in plotting spike number in response to incrementing steps of current pulses^27,28^. Changes in membrane potential (V_m_) were measured in the absence of any holding current.

### Drugs

Drugs were bath applied. Picrotoxin (PiTx) was purchased from Sigma. [4-(*N*-ethyl-*N*-phenylamino)-1,2-dimethyl-6-(methylamino) pyrimidinium chloride] (ZD-7288), D-(-)-2-Amino-5-phosphonopentanoic acid (D-AP5), 3,5-Dihydroxyphenylglycine (DHPG), (2 S)-2-Amino-2-[(1 S,2 S)-2-carboxycycloprop-1-yl]-3-(xanth-9-yl) propanoic acid (LY341495) and 2-methyl-6-(phenylethylyl)pyridine (MPEP) were purchased from Tocris Bioscience.

### Data acquisition and analysis

Recordings were obtained using an Axoclamp-2B (Molecular Devices) or a MultiClamp 700B (Molecular Devices) amplifier and pClamp10 software. Data were sampled at 10 kHz, filtered at 3 kHz, and digitized by a Digidata1322A (Molecular Devices). All data analyses were performed with custom written software in Igor Pro 6 (Wavemetrics).

Apparent input resistance was determined by the subtraction of the steady-state voltage change during hyperpolarizing current injection from the baseline.

Pooled data are presented as mean ± SEM. Statistical comparisons were made using Wilcoxon or Mann-Whitney test as appropriate with Sigma Plot software. Statistical correlations were tested using Spearman test. Data were considered as significant when p < 0.05.

### Modelling

A simple Hodgkin-Huxley-type model of hippocampal neuron was developed under LabView (LabView 7). The model had no dimension and included only the h conductance with parameters taken from Campanac *et al*., 2008. The leak resistance was set to 1 GΩ. The h-current was given by:

1 $${{\rm{I}}}_{{\rm{h}}}={{\rm{G}}}_{{\rm{h}}}\,\ast \,({{\rm{V}}}_{{\rm{m}}}-{{\rm{E}}}_{{\rm{h}}})$$

with V_m_ the membrane potential, E_h_ = −37.7 mV, and the h-conductance given by the following equation:

2 $${{\rm{G}}}_{{\rm{h}}}={{\rm{G}}}_{h,\max }\,\ast \,{\rm{n}}$$

The activation and deactivation time constants were determined by fitting experimental data from Campanac *et al*. (2008). The following differential equation was solved,

3 $${\rm{dn}}({\rm{V}},{\rm{t}})/{\rm{dt}}={{\rm{\alpha }}}_{{\rm{n}}}({\rm{V}})\,\ast \,[1-{\rm{n}}({\rm{V}},{\rm{t}})]$$

This equation corresponds to:

4 $${\rm{d}}({\rm{V}},{\rm{t}})/{\rm{dt}}=[{{\rm{n}}}_{\infty }({\rm{V}})-{\rm{n}}({\rm{V}},{\rm{t}})]/{\rm{\tau }}({\rm{V}})$$

5 $${{\rm{n}}}_{\infty }({\rm{V}})={{\rm{\alpha }}}_{{\rm{n}}}({\rm{V}})/[{{\rm{\alpha }}}_{{\rm{n}}}({\rm{V}})+{{\rm{\beta }}}_{{\rm{n}}}({\rm{V}})]$$

6 $${\rm{\tau }}({\rm{V}})=1\,{\rm{for}}\,{\rm{V}} > -30\,{\rm{mV}}$$

otherwise

7 $${\rm{\tau }}({\rm{V}})=1/[{{\rm{\alpha }}}_{{\rm{n}}}({\rm{V}})+{{\rm{\beta }}}_{{\rm{n}}}({\rm{V}})]$$

8 $${\alpha }_{{\rm{n}}}=0.0204/(1+\exp [({\rm{V}}+98.68)/13.24])$$

9 $${\beta }_{{\rm{n}}}=0.0176/(1+\exp [-({\rm{V}}+57.96)/13.2])$$

where n_∞_(V) is the steady-state activation parameter and τ(V) the activation time constant.

## Electronic supplementary material

Supplementary Information

## References

[1] Debanne D, Poo MM (2010). Spike-timing dependent plasticity beyond synapse - pre- and post-synaptic plasticity of intrinsic neuronal excitability. Front Synaptic Neurosci.

[2] Titley HK, Brunel N, Hansel C (2017). Toward a Neurocentric View of Learning. Neuron.

[3] Abraham WC, Gustafsson B, Wigstrom H (1987). Long-term potentiation involves enhanced synaptic excitation relative to synaptic inhibition in guinea-pig hippocampus. J Physiol.

[4] Daoudal G, Hanada Y, Debanne D (2002). Bidirectional plasticity of excitatory postsynaptic potential (EPSP)-spike coupling in CA1 hippocampal pyramidal neurons. Proc Natl Acad Sci USA.

[5] Campanac E, Debanne D (2008). Spike timing-dependent plasticity: a learning rule for dendritic integration in rat CA1 pyramidal neurons. J Physiol.

[6] Lopez-Rojas J, Heine M, Kreutz MR (2016). Plasticity of intrinsic excitability in mature granule cells of the dentate gyrus. Sci Rep.

[7] Campanac E (2013). Enhanced intrinsic excitability in basket cells maintains excitatory-inhibitory balance in hippocampal circuits. Neuron.

[8] Shim HG (2017). Long-Term Depression of Intrinsic Excitability Accompanied by Synaptic Depression in Cerebellar Purkinje Cells. J Neurosci.

[9] Turrigiano GG, Nelson SB (2004). Homeostatic plasticity in the developing nervous system. Nat Rev Neurosci.

[10] Desai NS, Rutherford LC, Turrigiano GG (1999). Plasticity in the intrinsic excitability of cortical pyramidal neurons. Nat Neurosci.

[11] Gasparini S, DiFrancesco D (1997). Action of the hyperpolarization-activated current (Ih) blocker ZD 7288 in hippocampal CA1 neurons. Pflugers Arch.

[12] Poolos NP, Migliore M, Johnston D (2002). Pharmacological upregulation of h-channels reduces the excitability of pyramidal neuron dendrites. Nat Neurosci.

[13] Fan Y (2005). Activity-dependent decrease of excitability in rat hippocampal neurons through increases in I(h). Nat Neurosci.

[14] Narayanan R, Johnston D (2007). Long-term potentiation in rat hippocampal neurons is accompanied by spatially widespread changes in intrinsic oscillatory dynamics and excitability. Neuron.

[15] Brager DH, Johnston D (2007). Plasticity of intrinsic excitability during long-term depression is mediated through mGluR-dependent changes in I(h) in hippocampal CA1 pyramidal neurons. J Neurosci.

[16] Wang Z, Xu NL, Wu CP, Duan S, Poo MM (2003). Bidirectional changes in spatial dendritic integration accompanying long-term synaptic modifications. Neuron.

[17] Campanac E, Daoudal G, Ankri N, Debanne D (2008). Downregulation of dendritic I(h) in CA1 pyramidal neurons after LTP. J Neurosci.

[18] Gastrein P (2011). The role of hyperpolarization-activated cationic current in spike-time precision and intrinsic resonance in cortical neurons *in vitro*. J Physiol.

[19] Dudek SM, Bear MF (1992). Homosynaptic long-term depression in area CA1 of hippocampus and effects of N-methyl-D-aspartate receptor blockade. Proc Natl Acad Sci USA.

[20] Mulkey RM, Malenka RC (1992). Mechanisms underlying induction of homosynaptic long-term depression in area CA1 of the hippocampus. Neuron.

[21] Debanne D, Gahwiler BH, Thompson SM (1994). Asynchronous pre- and postsynaptic activity induces associative long-term depression in area CA1 of the rat hippocampus *in vitro*. Proc Natl Acad Sci USA.

[22] Oliet SH, Malenka RC, Nicoll RA (1997). Two distinct forms of long-term depression coexist in CA1 hippocampal pyramidal cells. Neuron.

[23] Huber KM, Roder JC, Bear MF (2001). Chemical induction of mGluR5- and protein synthesis–dependent long-term depression in hippocampal area CA1. J Neurophysiol.

[24] Bashir ZI, Jane DE, Sunter DC, Watkins JC, Collingridge GL (1993). Metabotropic glutamate receptors contribute to the induction of long-term depression in the CA1 region of the hippocampus. Eur J Pharmacol.

[25] Zenke, F. & Gerstner, W. Hebbian plasticity requires compensatory processes on multiple timescales. *Philos Trans R Soc Lond B Biol Sci* **372** (2017). 10.1098/rstb.2016.0259 PMC5247595 28093557

[26] Breton JD, Stuart GJ (2009). Loss of sensory input increases the intrinsic excitability of layer 5 pyramidal neurons in rat barrel cortex. J Physiol.

[27] Gasselin C, Inglebert Y, Debanne D (2015). Homeostatic regulation of h-conductance controls intrinsic excitability and stabilizes the threshold for synaptic modification in CA1 neurons. J Physiol.

[28] Sourdet V, Russier M, Daoudal G, Ankri N, Debanne D (2003). Long-term enhancement of neuronal excitability and temporal fidelity mediated by metabotropic glutamate receptor subtype 5. J Neurosci.

[29] Karmarkar UR, Buonomano DV (2006). Different forms of homeostatic plasticity are engaged with distinct temporal profiles. Eur J Neurosci.

[30] Cudmore RH, Fronzaroli-Molinieres L, Giraud P, Debanne D (2010). Spike-time precision and network synchrony are controlled by the homeostatic regulation of the D-type potassium current. J Neurosci.

[31] Kuba H, Yamada R, Ishiguro G, Adachi R (2015). Redistribution of Kv1 and Kv7 enhances neuronal excitability during structural axon initial segment plasticity. Nat Commun.

[32] Honnuraiah S, Narayanan R (2013). A calcium-dependent plasticity rule for HCN channels maintains activity homeostasis and stable synaptic learning. PLoS One.

[33] Zenke F, Gerstner W, Ganguli S (2017). The temporal paradox of Hebbian learning and homeostatic plasticity. Curr Opin Neurobiol.

[34] Debanne D, Daoudal G, Sourdet V, Russier M (2003). Brain plasticity and ion channels. J Physiol Paris.

[35] Shah MM (2014). Cortical HCN channels: function, trafficking and plasticity. J Physiol.

[36] Shin M, Chetkovich DM (2007). Activity-dependent regulation of h channel distribution in hippocampal CA1 pyramidal neurons. J Biol Chem.

[37] Luthi A, McCormick DA (1999). Modulation of a pacemaker current through Ca(2+)-induced stimulation of cAMP production. Nat Neurosci.

[38] Santoro B, Wainger BJ, Siegelbaum SA (2004). Regulation of HCN channel surface expression by a novel C-terminal protein-protein interaction. J Neurosci.

[39] Lewis AS (2009). Alternatively spliced isoforms of TRIP8b differentially control h channel trafficking and function. J Neurosci.

[40] Santoro B (2009). TRIP8b splice variants form a family of auxiliary subunits that regulate gating and trafficking of HCN channels in the brain. Neuron.

[41] Zolles G (2009). Association with the auxiliary subunit PEX5R/Trip8b controls responsiveness of HCN channels to cAMP and adrenergic stimulation. Neuron.

[42] Santoro B (2011). TRIP8b regulates HCN1 channel trafficking and gating through two distinct C-terminal interaction sites. J Neurosci.

[43] Huber KM, Kayser MS, Bear MF (2000). Role for rapid dendritic protein synthesis in hippocampal mGluR-dependent long-term depression. Science.

[44] Glanzer J (2005). RNA splicing capability of live neuronal dendrites. Proc Natl Acad Sci USA.

[45] Brager DH, Lewis AS, Chetkovich DM, Johnston D (2013). Short- and long-term plasticity in CA1 neurons from mice lacking h-channel auxiliary subunit TRIP8b. J Neurophysiol.

[46] van Welie I, van Hooft JA, Wadman WJ (2004). Homeostatic scaling of neuronal excitability by synaptic modulation of somatic hyperpolarization-activated Ih channels. Proc Natl Acad Sci USA.

