# Supplementary material for: Plasticity of intrinsic excitability during LTD is mediated by bidirectional changes in h-channel activity
Source: Sci Rep. 2017 Oct 31;7:14418. doi: 10.1038/s41598-017-14874-z (PMC5663755; doi:10.1038/s41598-017-14874-z)
Supplement: Supplementary file 1 — Supplementary Information [file 41598_2017_14874_MOESM1_ESM.docx]

**Supplementary Information**

Plasticity of intrinsic excitability during LTD is mediated by bidirectional changes in h-channel activity

Célia Gasselin^1^, Yanis Inglebert^1^, Norbert Ankri & Dominique Debanne^2^

UNIS, INSERM UMR_S 1072, Aix-Marseille Université, 13015 Marseille, France

*^1^These authors equally contributed to the work*

^2^Corresponding author

[*dominique.debanne@inserm.fr*](mailto:dominique.debanne@inserm.fr)


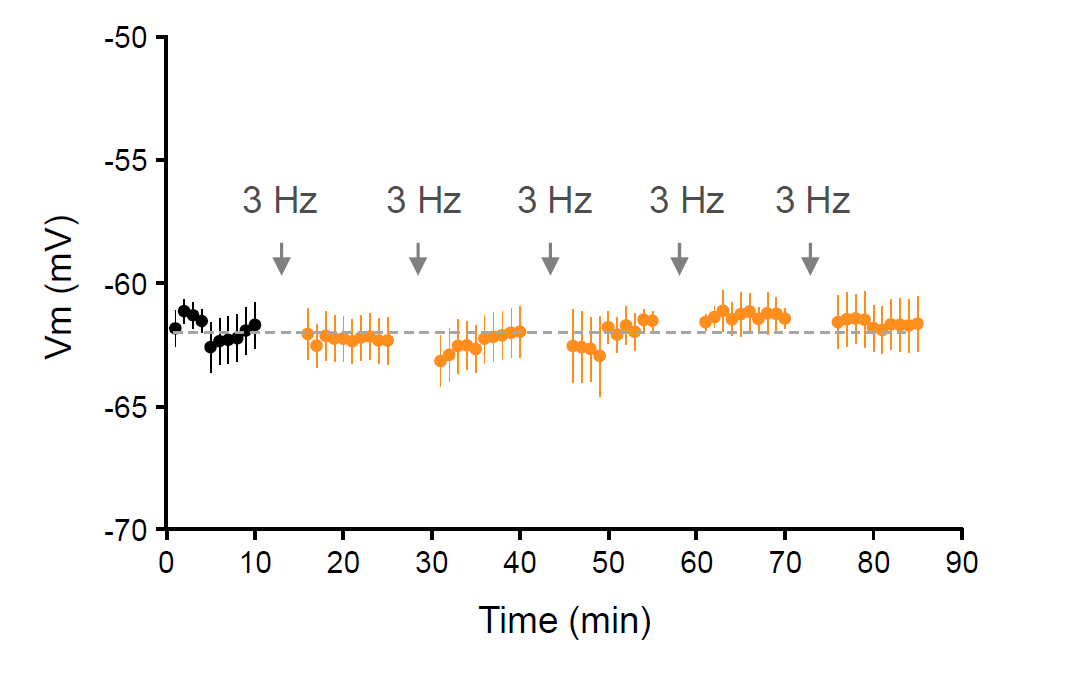


**Supplementary Figure 1.** Analysis of V_m_. Time –course of V_m_ in the experiment illustrated in Fig.1C. No significant change in V_m_ occurs following the different episodes of 3 Hz stimulation.


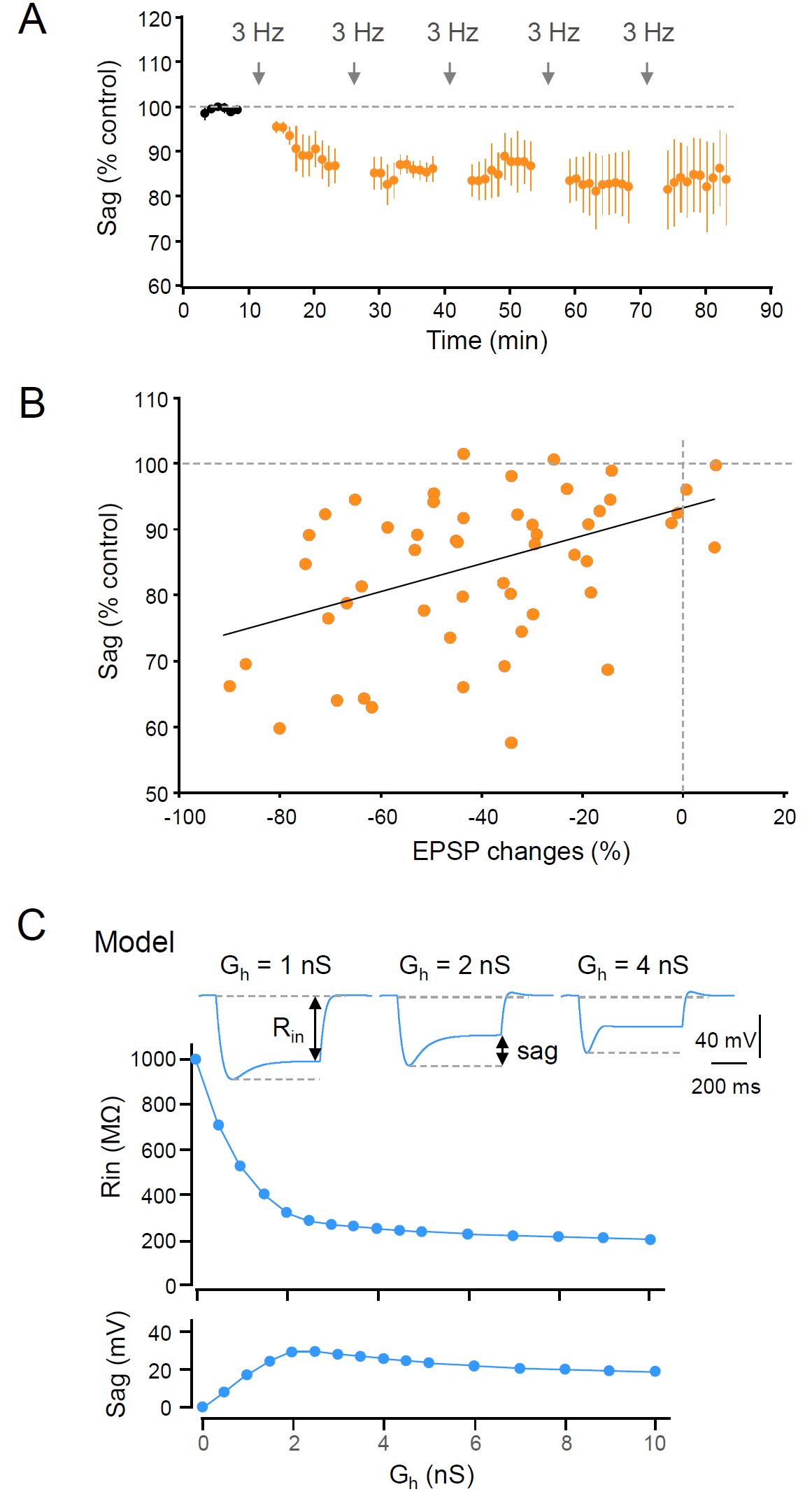


**Supplementary Figure 2.** Analysis of the sag. **A**, Time-course of the sag in the experiment illustrated in Fig. 1C. **B**, Plot of sag as a function of EPSP change. Note the correlation (y=0.213 x + 93.1; r=0.44). **C**, Model of h-current. Top, voltage traces obtained by injection of -120 pA in the model with G_h_ = 1, 2 or 4 nS. While R_in_ diminishes with increased G_h_, the sag is reduced at 4 nS compared to 2 nS. Middle, R_in_ as a function of G_h_. Bottom, sag as a function of G_h_.


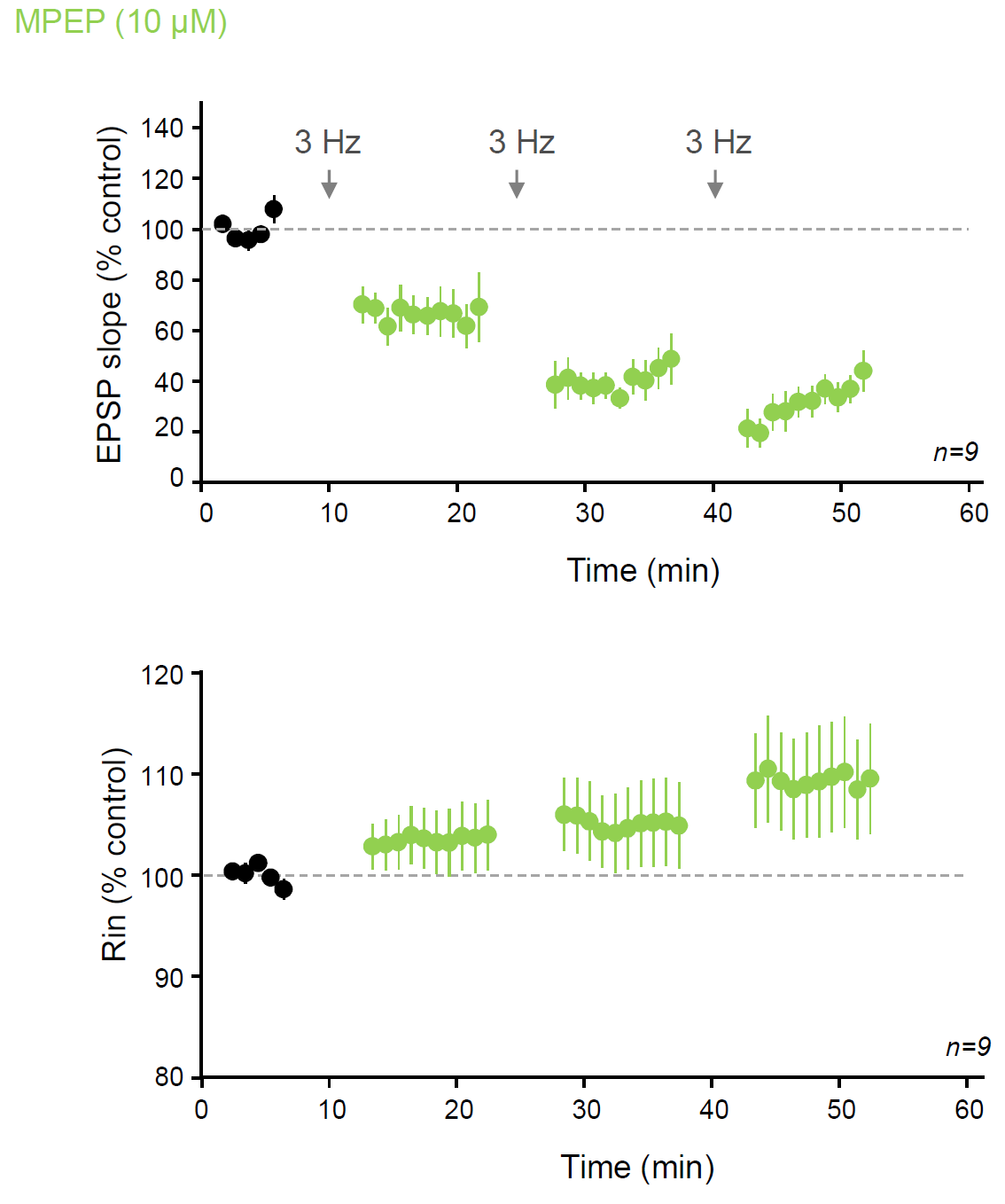


**Supplementary Figure 3.** LTD induced in the presence of the mGluR5 antagonist, MPEP is still associated with an elevated R_in_. Top, time course of synaptic changes induced by 3 Hz stimulation in the presence of 10 µM MPEP. Bottom, changes in R_in_ in the same experiments. Note the increase in R_in_ after the first episode of 3 Hz stimulation.
